# High prevalence of hyposalivation in individuals with neurofibromatosis 1: a case–control study

**DOI:** 10.1186/s13023-015-0239-4

**Published:** 2015-02-28

**Authors:** Karin Soares Cunha, Rafaela Elvira Rozza-de-Menezes, Eloá Borges Luna, Lilian Machado de Sousa Almeida, Raquel Richelieu Lima de Andrade Pontes, Paula Nascimento Almeida, Letícia Vidaurre de Aguiar, Eliane Pedra Dias

**Affiliations:** Postgraduate Program in Pathology, School of Medicine, Universidade Federal Fluminense, Niterói, RJ Brazil; Neurofibromatosis National Center (Centro Nacional de Neurofibromatose), Rio de Janeiro, RJ Brazil; School of Dentistry, Universidade Federal Fluminense, Niterói, RJ Brazil; Department of Pathology, School of Medicine, Universidade Federal Fluminense, Niterói, RJ Brazil

**Keywords:** Neurofibromatosis 1, Salivary glands, Xerostomia, Oral manifestations

## Abstract

**Background:**

Neurofibromatosis type 1 (NF1) is one of the most common genetic diseases in humans and has widely variable expressivity. Oral manifestations are common, but there are no studies that investigated functional alterations in salivary glands in NF1. Our aim was to evaluate the salivary flow rate in NF1 individuals, comparing to a control group, and to investigate the possible causes and some consequences of salivary gland alteration.

**Methods:**

This is a case–control study that evaluated the salivary flow rate of NF1 individuals (n = 49) and compared to an age and sex-matched control group. We have also investigated the possible causes and consequences of hyposalivation in NF1 individuals through anamnesis, a specific questionnaire, physical examination, tongue coating evaluation and cytopathological exam to assess the prevalence of oral candidiasis.

**Results:**

Hyposalivation at rest was present in 59% (29/49) of NF1 individuals in contrast to 22% (11/49) in the control group, being statistically significant (*P* <0.0001; Wilcoxon rank-sum test). The analysis of the adjusted residual showed that the prevalence of hyposalivation in NF1 individuals (46.9%) was 4-fold higher than in controls (10.2%). None of the possible causes of hyposalivation (medications, low liquid intake, caffeinated or stimulant drink use, mouth breathers, alcohol, smoke and plexiform neurofibroma close to or involving major salivary glands areas) had important impact on the salivary flow rate in NF1 individuals.

**Conclusions:**

Hyposalivation may be a consequence of NF1, as occurs in other genetic diseases. More studies are necessary to understand if there is and what is the relationship between NF1 and hyposalivation.

**Electronic supplementary material:**

The online version of this article (doi:10.1186/s13023-015-0239-4) contains supplementary material, which is available to authorized users.

## Background

Neurofibromatosis 1 (NF1, OMIM 162200) is one of the most common genetic diseases and has widely variable expressivity [1]. Oral manifestations are common, [2-6] but there are no studies that investigated functional alterations in salivary glands in NF1. Our aim was to evaluate the salivary flow rate in NF1 individuals, comparing to a control group, and to investigate the possible causes and some consequences of salivary gland alteration.

## Methods

This case–control study was approved by the Ethics Committee (#31640/2012) and conducted at Oral Diagnosis Ambulatory and Pathological Anatomy Service of Antônio Pedro University Hospital of Universidade Federal Fluminense, Brazil.

The study group was composed of 49 NF1 individuals, according to clinical criteria [7]. To compare the sialometry values of NF1 individuals, a sex and age-matched control group (n=49) composed by non-NF1 individuals was included. Data about smoking, alcohol use, and intake of hyposalivation-inducing drugs was obtained from both groups.

Sialometry (unstimulated whole saliva flow rate) was performed between 8:00–11:30 AM and the participants did not ingest food or liquids 1 hour before the exam. After discarding all the saliva present in the mouth, saliva was collected for 5 minutes without stimulus. The salivary flow rate (mL/min) was calculated and classified: normal: ≥0.3; low: ≥0.1 and <0.3; very low: <0.1.

Tongue coating was evaluated according to previous studies [8,9], using digital photographs. The tongue dorsum area was divided into nine equal sections and each section was assigned a value: 0 = no; 1 = thin, 2 = thick coating. The sum of the results of each section was calculated, divided by 18 and multiplied by 100 to achieve the tongue coating index (TCI) percentage score.

Oral candidiasis was investigated by clinical and cytopathology exam through scrapping the tongue dorsum with a cytobrush. Glass slides stained with Papanicolaou and Periodic Acid-Schiff were analyzed according to specific criteria [10].

NF1 participants also answered to a questionnaire (Table 1).

**Table 1:** **Questionnaire used in the study group (with NF1)**

**Questions**	**Response**	
**Oral Dryness Questionnaire**	Yes	No
1. Do you fell dry oral mucosa sensation?	Yes	No
2. Do you feel dry lips sensation?	Yes	No
3. Do you have difficult to swallow dry food?	Yes	No
4. Do you drink liquids to aid swallowing dry food?	Yes	No
5. Do you feel change in saliva viscosity?	Yes	No
6. Do you feel a decreased amount of saliva in your mouth?	Yes	No
7. Do you feel enough or increased amount of saliva in your mouth?	Yes	No
**Other Questions**	Yes	No
8. Do you intake at least 2 liters of liquid daily?	Yes	No
9. Do you commonly drink caffeinated or stimulant drinks?	Yes	No
10. You are a mouth breather?	Yes	No

## Results

Additional files 1, 2 and 3 show details of the results. Table 2 summarizes the sample characteristics. There was no heterogeneity between the study and control group according to smoking, alcohol use and intake of medications that could cause hyposalivation. Four (8%) NF1 participants had plexiform neurofibromas close to or involving the major salivary glands areas and only one (25%) had hyposalivation.

**Table 2:** **Summary of the sample characteristics**

**Data**	**Study group (with NF1)**	**Control group**	***P*** *
Number	49	49	
Age (years)	42.5 ± 14.8^†^	42.5 ± 14.8^†^	–
Sex (female/male)	35/14 (2.5:1.0)	35/14 (2.5:1.0)	–
Smoker	4 (8%)	9 (18%)	0.227
Alcohol user	8 (16%)	10 (20%)	0.774
Hyposalivation-inducing drugs	14 (28%)	18 (36%)	0.344

NF1, Neurofibromatosis 1.

*P value is from McNemar χ^2^ test; ^†^ Results indicate mean ± standard deviation.

In NF1 individuals, 59% (29/49) had hyposalivation; 32% (16/49) had severe hyposalivation. In the control group, 22% (11/49) of individuals had hyposalivation; 8% (4/49) presented severe hyposalivation. There was a statistically significant difference between the sialometry values of the study and control group (*P* < 0.0001; Wilcoxon rank-sum test). There was a statistically significant association between NF1 individuals and presence of hyposalivation (*P* = 0.001; McNemar’s χ^2^ test). The analysis of the adjusted residual showed that the prevalence of hyposalivation in NF1 individuals (46.9%) was 4-fold higher than in controls (10.2%).

Forty-six NF1 participants answered to the questionnaire. The number of positive answers per individual to the oral dryness questions was assessed to investigate them as possible predictor of hyposalivation. The results are shown in Tables 3–4.

**Table 3:** **Hyposalivation and oral dryness questionnaire in study group (with NF1)**

**Oral Dryness Assessment Questionnaire**	**UWSFR**		
	**Hyposalivation (%)**	**OR (IC 95%)**	***P*** *
1. Do you fell dry oral mucosa sensation?	9 (18)	1.8 (0.4 – 7.3)	0.51
2. Do you feel dry lips sensation?	10 (20)	0.8 (0.2 – 2.6)	0.76
3. Do you have difficult to swallow dry food?	6 (12)	1.5 (0.3 – 7)	0.71
4. Do you drink liquids to aid swallowing dry food?	14 (28)	1.8 (0.5 – 6.1)	0.37
5. Do you feel change in saliva viscosity?	11 (22)	1.9 (0.5 – 6.9)	0.36
6. Do you feel a decreased amount of saliva in your mouth?	8 (16)	0.9 (0.2 – 3.2)	0.88
7. Do you feel enough or increased amount of saliva in your mouth?	3 (6)	0.4 (0.09 – 2)	0.42

UWSFR, unstimulated whole saliva flow rate; OR (IC 95%), Odds ratio and 95% of confidence interval.

*P value is from Qui-Square analysis or Fisher’s exact test.

**Table 4:** **Score of positive answers from oral dryness assessment questionnaire according to hyposalivation in study group (with NF1)**

Number of positive answers*	**UWSFR**		
	**Hyposalivation (%)**	**OR (IC 95%)**	***P*** **
None	7 (14)	0.9 (0.2 – 3.7)	0.97
One	8 (16.3)	0.6 (0.1 – 3)	0.7
Two	4 (8.1)	0.4 (0.1 – 2.1)	0.45
Three	7 (14)	2.9 (0.5 – 16.2)	0.27
Four	2 (4)	1.7 (1.3 – 2.2)	0.5
Five	2 (4)	1.4 (0.1 – 17.1)	1.0
Six	2 (4)	0.6 (0.8 – 5.3)	1.0

UWSFR, Unstimulated whole saliva flow rate; OR (IC 95%), Odds ratio and 95% of confidence interval.

*Considering the first six questions of the Oral Dryness Questionnaire, **P value is from Qui-Square analysis or Fisher’s exact test.

Oral candidiasis was present in 22% (11/49) of NF1 individuals. Of these, 54% (6/49) had no clinical signs or symptoms of candidiasis, 36% (3/49) had erythematous and 9% (1/49) pseudomembranous candidiasis.

Fifty-five percent (27/49) of NF1 individuals had more than 50% and 16% had more than 80% of the tongue covered by coating. The mean of TCI was 50% (standard deviation = 26.7, median = 50, first quartile = 25, third quartile = 72).

Any of the investigated variables were associated with hyposalivation in NF1 individuals (Table 5).

**Table 5:** **Correlation between hyposalivation and its causes and consequences in study group (with NF1)**

**Variables**	**Hyposalivation**		
	**UWSFR**	**OR (95% IC)**	***P*** *
Age	43 ± 15.6^†^	–	0.77
Female/Male	23/6 (3.8/1)	2.5 (0.7-9.0)	0.14
Plexiform neurofibroma close to or involving major salivary glands areas	1 (3.4%)	0.2 (0.01-2.1)	0.29
Smoker	1 (3.4%)	0.2 (0.01-2.1)	0.29
Alcohol user	5 (17%)	1.1 (0.2-5.6)	1.0
Hyposalivation-inducing drugs	15 (51%)	1.6 (0.5-5.0)	0.41
Intake less than 2 liters of liquid daily	8 (27%)	1.7 (0.5-5.6)	0.36
Caffeinated or stimulant drink use	13 (44%)	1.2 (0.3-4.1)	0.68
Mouth breather	14 (48%)	1.4 (0.4-4.8)	0.51
Oral candidiasis	7 (24%)	1.2 (0.3-5.0)	1.0
Tongue coating index	44.4 ± 27.3^†^	–	0.05

UWSFR, Unstimulated whole saliva flow rate; OR (95% IC), Odds ratio and 95% of confidence interval.

*P value is from Qui-square analysis and Fisher’s exact tests for categorical variables and Mann–Whitney test for numerical variables; ^†^Results of age and tongue coating index indicate mean ± standard deviation.

## Discussion

We showed that NF1 individuals present high prevalence (59%) of hyposalivation comparing to a control group (*P* < 0.001), with a proportion 4-fold higher than the controls.

Xerostomia often occurs when sialometry value is reduced by about 50% [11]. Due to this lack of correlation between xerostomia and hyposalivation, some authors have investigated whether positive answers to other questions related to the dry mouth symptoms have correlation with sialometry values [11]. In our study, hyposalivation was not associated with xerostomia in NF1 individuals, neither with other questions from the oral dryness questionnaire.

Xerostomia can also occur in individuals with normal sialometry values [11,12]. It can occur in mouth breathers or because of changes in the sialochemistry, viscoelastic properties of saliva or by sensory alterations. In the present study, there was no NF1 individual who complained of xerostomia and did not present hyposalivation.

Saliva exerts many functions in oral cavity, including the control of the composition of the microflora. We found 22% of NF1 participants with cytological evidence of oral candidiasis and 55% with 50% of the tongue covered by coating, but none were associated with hyposalivation.

None of the investigated causes of hyposalivation (medications, low liquid intake, caffeinated or stimulant drink use, alcohol, and smoke) were associated with low salivary flow rate in NF1. Moreover, only 4 (8%) NF1 individuals had plexiform neurofibromas in areas of major salivary glands, which could cause atrophy of the acinar cells by compression or infiltration by the tumor cells, and just one of them had hyposalivation.

Alterations in the salivary glands (acini and ducts) caused by mutations in the *NF1* gene may be a possible explanation for the high prevalence of hyposalivation in NF1 individuals. Neurofibromin is a negative regulator of the Ras pathways and there is a complex signaling cross-talks between neurofibromin and other members of the superfamily of small GTPases, including Rho binding domain [13]. Rho family of small GTPases presents a crucial role in lumen morphogenesis of salivary glands in animal models and in acinus formation in human salivary gland cell line [13-16]. Kimura et al. [17] showed that neurofibromin is strongly expressed in ductal cells of the parotids. Other salivary glands were not evaluated in that study.

Moreover, the volume and type of saliva is controlled by autonomic nervous system and the blood supply to the glands influences the salivary secretion. Since neurofibromin is expressed in peripheral and central nervous system, as well as in blood vessel smooth muscle and endothelial cells [18-20], alteration in autonomic nervous system and in blood flux to the salivary glands may also be involved in hyposalivation in NF1 individuals.

## Conclusions

Hyposalivation may be a consequence of NF1, as occurs in other genetic diseases. More studies are necessary to understand if there is and what is the relationship between NF1 and hyposalivation.

## Availability of supporting data

The data sets supporting the results of this article are included within the paper and its additional files.

## Additional files

Additional file 1:
**Details of the clinical data of the study group (NF1 group).**
Additional file 2:
**Details of the clinical data of the control group.**
Additional file 3:
**Details of the results of the oral dryness assessment questionnaire (NF1 group).**

## Abbreviations

NF1: Neurofibromatosis 1
TCI: Tongue coating index
UWSFR: Unstimulated whole saliva flow rate
OR: Odds ratio
IC: Confidence interval
