# Supplementary material for: High prevalence of hyposalivation in individuals with neurofibromatosis 1: a case–control study
Source: Orphanet J Rare Dis. 2015 Feb 28;10:24. doi: 10.1186/s13023-015-0239-4 (PMC4351927; doi:10.1186/s13023-015-0239-4)
Supplement: Additional file 1: — Details of the clinical data of the study group (NF1 group). [file 13023_2015_239_MOESM1_ESM.pdf]

Additional file 1 – Details of the clinical data of the study group (NF1 group)

Code	Age	Sex	Plexiform neurofibroma close to or involving major salivary glands areas	Smoker	Alcohol user	Hyposalivation -inducing drugs	Intake less than 2 liters of liquid daily	Caffeinated or stimulant drink use	Mouth breather	UWSFR mL/min	UWSFR	Oral candidiasis	TCI	TCI (%)
1	46	F	Yes	No	No	No	No	No	No	0.4	Normal	No	7	38
2	45	M	No	No	No	No	Yes	Yes	Yes	0.48	Normal	No	7	38
3	67	F	No	No	No	Yes	Yes	No	No	0.02	Very low	No	4	22
4	35	F	Yes	No	No	Yes	No	No	Yes	0.64	Normal	Yes	10	55
5	71	F	No	No	Yes	Yes	No	No	No	0.12	Low	No	5	27
6	23	F	No	No	No	No	No	No	No	0.04	Very low	No	0	0
7	52	F	No	No	No	No	No	Yes	No	0.64	Normal	No	13	72
8	46	F	No	No	No	Yes	No	Yes	Yes	0.24	Low	No	2	11
9	35	M	No	No	No	No	Yes	Yes	No	0.24	Low	No	8	44
10	43	M	No	No	No	No	Yes	Yes	No	0.02	Very low	No	13	72
11	44	F	Yes	No	No	No	Yes	No	Yes	0.1	Low	No	11	61
12	54	F	No	No	No	No	Yes	Yes	No	0.04	Very low	Yes	1	6
13	67	F	No	No	Yes	Yes	No	No	No	0.02	Very low	Yes	7	38
14	37	F	No	No	No	No	No	No	No	0.4	Normal	No	15	83
15	58	F	No	No	No	Yes	Yes	No	Yes	0.24	Low	No	12	66
16	31	F	No	Yes	No	Yes	Yes	Yes	Yes	0.6	Normal	No	6	33
17	26	F	No	No	No	No	No	Yes	Yes	1.2	Normal	No	7	38
18	41	F	No	No	Yes	No	No	Yes	Yes	0.07	Very low	No	10	55
19	56	F	No	No	No	No	No	No	Yes	0.04	Very low	Yes	3	16
20	45	F	No	No	No	Yes	No	No	No	0.02	Very low	Yes	12	66
21	55	F	No	Yes	Yes	Yes	No	No	Yes	0.6	Normal	Yes	10	55
22	66	M	No	No	Yes	Yes	Yes	Yes	Yes	0.4	Normal	Yes	15	83
23	54	F	No	No	No	Yes	No	No	Yes	0.04	Very low	Yes	18	100
24	44	F	No	No	No	Yes	Yes	Yes	Yes	0.2	Low	No	6	33
25	37	F	No	No	No	Yes	No	Yes	Yes	0	Very low	No	2	11
26	31	M	No	No	Yes	No	NE	NE	NE	0.04	Very low	No	3	16
27	18	M	No	No	No	No	No	No	No	0.4	Normal	No	16	88
28	15	F	No	No	No	Yes	No	No	No	0.16	Low	No	4	22
29	37	M	No	No	No	No	NE	NE	NE	0.2	Low	No	15	83
30	42	F	No	No	No	Yes	No	Yes	Yes	0.12	Low	Yes	14	77
31	64	F	No	No	No	Yes	No	No	No	0.04	Very low	Yes	11	61
32	47	M	No	Yes	Yes	No	No	No	No	0.4	Normal	No	14	77
33	39	F	No	No	Yes	Yes	Yes	No	No	0.24	Low	No	12	66
34	46	F	No	No	No	No	Yes	No	Yes	0.02	Very low	No	15	83
35	28	F	Yes	No	No	No	Yes	Yes	No	1	Normal	No	6	33

**36**	15	F	No	No	No	No	Yes	Yes	Yes	0.24	Low	No	9	50
**37**	22	M	No	No	No	No	Yes	Yes	No	0.08	Very low	No	9	50
**38**	54	F	No	No	No	No	Yes	No	No	0.8	Normal	No	14	77
**39**	21	M	No	No	No	No	Yes	Yes	Yes	0.24	Low	No	4	22
**40**	34	F	No	No	No	Yes	No	Yes	Yes	0.8	Normal	No	15	83
**41**	48	M	No	No	No	Yes	Yes	No	No	0.4	Normal	No	15	83
**42**	41	F	No	No	No	No	Yes	Yes	No	0.2	Low	No	13	72
**43**	29	M	No	No	No	No	No	Yes	Yes	0.68	Normal	Yes	2	11
**44**	75	M	No	No	No	No	Yes	No	No	0.8	Normal	No	13	72
**45**	39	M	No	No	No	No	No	No	No	0.4	Normal	No	3	16
**46**	41	F	No	No	No	Yes	Yes	No	No	1	Normal	No	13	72
**47**	62	F	No	No	No	Yes	Yes	No	Yes	0.2	Low	No	4	22
**48**	29	F	No	No	No	Yes	NE	NE	NE	0.4	Normal	No	9	50
**49**	28	F	No	Yes	Yes	Yes	Yes	Yes	Yes	0.2	Low	No	5	27

UWSFR: Unstimulated whole saliva flow rate; mL/min: milliliters per minute; TCI: Tongue Coating Index
