# Supplementary material for: High prevalence of hyposalivation in individuals with neurofibromatosis 1: a case–control study
Source: Orphanet J Rare Dis. 2015 Feb 28;10:24. doi: 10.1186/s13023-015-0239-4 (PMC4351927; doi:10.1186/s13023-015-0239-4)
Supplement: Additional file 2: — Details of the clinical data of the control group. [file 13023_2015_239_MOESM2_ESM.pdf]

Additional file 2 – Details of the clinical data of the control group

Code	Age	Sex	Smoker	Alcohol user	Hyposalivation-inducing drugs	UWSFR mL/min	UWSFR
**1**	48	F	No	No	No	0.9	Normal
**2**	44	M	No	No	No	1	Normal
**3**	67	F	No	No	Yes	0.16	Low
**4**	34	F	Yes	No	No	0.2	Low
**5**	71	F	No	No	Yes	0.8	Normal
**6**	24	F	No	No	Yes	0.8	Normal
**7**	53	F	No	No	Yes	0.16	Low
**8**	46	F	No	No	Yes	1	Normal
**9**	37	M	Yes	No	No	0.4	Normal
**10**	43	M	Yes	Yes	No	0.25	Low
**11**	45	F	No	No	No	0.4	Normal
**12**	54	F	No	No	Yes	0.04	Very Low
**13**	65	F	No	No	Yes	0.4	Normal
**14**	38	F	No	No	No	0.8	Normal
**15**	60	F	No	No	Yes	1	Normal
**16**	31	F	Yes	No	No	0.6	Normal
**17**	28	F	No	No	No	1.1	Normal
**18**	41	F	No	No	No	0	Very low
**19**	56	F	No	No	Yes	0.32	Normal
**20**	45	F	No	Yes	No	0.27	Low
**21**	55	F	No	Yes	Yes	0.2	Low
**22**	66	M	No	Yes	Yes	1.2	Normal
**23**	53	F	Yes	Yes	No	0.42	Normal
**24**	43	F	Yes	Yes	No	0.8	Normal
**25**	38	F	No	No	Yes	1	Normal
**26**	32	M	No	Yes	No	2	Normal
**27**	16	M	No	No	No	0.3	Normal
**28**	15	F	No	No	No	0.5	Normal
**29**	36	M	No	No	No	1	Normal
**30**	39	F	No	No	No	0.4	Normal
**31**	63	F	Yes	No	Yes	1.4	Normal
**32**	45	M	No	No	No	0.16	Low
**33**	39	F	No	No	No	0.8	Normal
**34**	47	F	No	No	Yes	0.7	Normal
**35**	29	F	No	No	No	0.4	Normal
**36**	14	F	No	No	No	0.4	Normal

**37**	24	M	Yes	No	No	1	Normal
**38**	54	F	No	No	Yes	0.5	Normal
**39**	23	M	No	No	No	0.3	Normal
**40**	35	F	No	No	No	0.9	Normal
**41**	48	M	Yes	Yes	No	0	Very Low
**42**	41	F	No	No	No	2	Normal
**43**	26	M	No	Yes	No	1.1	Normal
**44**	77	M	No	Yes	Yes	0.5	Normal
**45**	40	M	No	No	No	0.8	Normal
**46**	41	F	No	No	No	0.4	Normal
**47**	62	F	No	No	Yes	0.4	Normal
**48**	29	F	No	No	No	1.2	Normal
**49**	28	F	No	No	Yes	0.02	Very low

F: Female; M: Male; UWSFR: Unstimulated whole salivar flow rate; NE: Not evaluated
