# Supplementary material for: High prevalence of hyposalivation in individuals with neurofibromatosis 1: a case–control study
Source: Orphanet J Rare Dis. 2015 Feb 28;10:24. doi: 10.1186/s13023-015-0239-4 (PMC4351927; doi:10.1186/s13023-015-0239-4)
Supplement: Additional file 3: — Details of the results of the oral dryness assessment questionnaire (NF1 group). [file 13023_2015_239_MOESM3_ESM.pdf]

Additional file 3 – Details of the results of the oral dryness assessment questionnaire (NF1 group)

| XEROSTOMIA ASSESSMENT QUESTIONNAIRE | | | | | | | | | | | | | | |
|---|---|---|---|---|---|---|---|---|---|---|---|---|---|---|
| **Code** | Question 1 | Question 2 | Question 3 | Question 4 | Question 5 | Question 6 | Question 7 | Score 1 | Score 2 | Score 3 | Score 4 | Score 5 | Score 6 | No positive answer |
| **1** | No | No | No | No | No | No | No | No | No | No | No | No | No | **Yes** |
| **2** | No | No | No | **Yes** | **Yes** | No | **Yes** | No | No | **Yes** | No | No | No | No |
| **3** | No | No | **Yes** | **Yes** | **Yes** | No | No | No | No | **Yes** | No | No | No | No |
| **4** | **Yes** | **Yes** | **Yes** | **Yes** | **Yes** | **Yes** | No | No | No | No | No | No | **Yes** | No |
| **5** | **Yes** | No | No | **Yes** | No | No | No | No | No | **Yes** | No | No | No | No |
| **6** | **Yes** | **Yes** | No | **Yes** | **Yes** | No | **Yes** | No | No | No | No | **Yes** | No | No |
| **7** | No | No | No | No | No | No | No | No | No | No | No | No | No | **Yes** |
| **8** | **Yes** | **Yes** | **Yes** | **Yes** | **Yes** | **Yes** | No | No | No | No | No | No | **Yes** | No |
| **9** | No | No | No | No | **Yes** | No | No | **Yes** | No | No | No | No | No | No |
| **10** | No | No | No | No | No | No | No | No | No | No | No | No | No | **Yes** |
| **11** | No | **Yes** | No | No | **Yes** | No | No | No | **Yes** | No | No | No | No | No |
| **12** | **Yes** | No | No | **Yes** | No | **Yes** | No | No | No | **Yes** | No | No | No | No |
| **13** | No | No | No | No | No | No | No | No | No | No | No | No | No | **Yes** |
| **14** | No | No | No | No | No | No | No | No | No | No | No | No | No | **Yes** |
| **15** | **Yes** | **Yes** | No | **Yes** | No | **Yes** | No | No | No | No | **Yes** | No | No | No |
| **16** | **Yes** | No | No | **Yes** | No | No | No | No | **Yes** | No | No | No | No | No |
| **17** | No | **Yes** | No | No | No | No | No | **Yes** | No | No | No | No | No | No |
| **18** | **Yes** | No | No | No | **Yes** | No | No | No | **Yes** | No | No | No | No | No |
| **19** | No | No | No | No | No | No | No | No | No | No | No | No | No | **Yes** |
| **20** | No | **Yes** | No | No | **Yes** | **Yes** | No | No | No | **Yes** | No | No | No | No |
| **21** | No | **Yes** | No | No | No | **Yes** | No | **Yes** | **Yes** | No | No | No | No | No |
| **22** | No | No | **Yes** | No | No | No | No | **Yes** | No | No | No | No | No | No |
| **23** | **Yes** | **Yes** | **Yes** | **Yes** | No | **Yes** | No | No | No | No | No | **Yes** | No | No |
| **24** | No | No | No | **Yes** | No | No | No | No | No | No | No | No | No | No |
| **25** | No | No | No | **Yes** | **Yes** | No | **Yes** | No | No | **Yes** | No | No | No | No |
| **26** | NE | NE | NE | NE | NE | NE | NE | NE | NE | NE | NE | NE | NE | NE |
| **27** | No | **Yes** | No | No | No | **Yes** | No | No | **Yes** | No | No | No | No | No |
| **28** | No | No | No | **Yes** | No | No | No | **Yes** | No | No | No | No | No | No |
| **29** | NE | NE | NE | NE | NE | NE | NE | NE | NE | NE | NE | NE | NE | NE |
| **30** | No | No | No | No | **Yes** | **Yes** | No | No | **Yes** | No | No | No | No | No |
| **31** | No | **Yes** | **Yes** | No | **Yes** | No | No | No | No | **Yes** | No | No | No | No |
| **32** | No | No | No | No | No | No | No | No | No | No | No | No | No | **Yes** |
| **33** | No | No | No | No | No | No | No | No | No | No | No | No | No | **Yes** |
| **34** | **Yes** | **Yes** | No | **Yes** | No | **Yes** | No | No | No | No | **Yes** | No | No | No |
| **35** | No | **Yes** | No | No | No | **Yes** | No | No | **Yes** | No | No | No | No | No |
| **36** | No | No | No | No | No | No | No | No | No | No | No | No | No | **Yes** |
| **37** | No | No | No | No | No | No | No | No | No | No | No | No | No | **Yes** |

| | | | | | | | | | | | | | | |
|---|---|---|---|---|---|---|---|---|---|---|---|---|---|---|
| **38** | No | **Yes** | No | **Yes** | No | No | **Yes** | No | No | **Yes** | No | No | No | No |
| **39** | **Yes** | **Yes** | **Yes** | **Yes** | **Yes** | **Yes** | No | No | No | No | No | No | **Yes** | No |
| **40** | **Yes** | **Yes** | No | **Yes** | **Yes** | **Yes** | No | No | No | No | No | **Yes** | No | No |
| **41** | No | No | No | **Yes** | **Yes** | No | No | No | **Yes** | No | No | No | No | No |
| **42** | No | No | No | No | No | No | No | No | No | No | No | No | No | **Yes** |
| **43** | **Yes** | **Yes** | **Yes** | **Yes** | **Yes** | **Yes** | No | No | No | No | No | No | **Yes** | No |
| **44** | No | No | No | No | No | No | **Yes** | **Yes** | No | No | No | No | No | No |
| **45** | No | No | No | No | No | No | **Yes** | **Yes** | No | No | No | No | No | No |
| **46** | No | No | No | No | No | No | No | No | No | No | No | No | No | **Yes** |
| **47** | No | No | **Yes** | **Yes** | No | No | No | No | **Yes** | No | No | No | No | No |
| **48** | NE | NE | NE | NE | NE | NE | NE | NE | NE | NE | NE | NE | NE | NE |
| **49** | No | **Yes** | No | **Yes** | No | No | **Yes** | No | No | **Yes** | No | No | No | No |

Question 1: Do you fell dry oral mucosa sensation?; Question 2: Do you feel dry lips sensation?; Question 3: Do you have difficult to swallow dry food?; Question 4: Do you drink liquids to aid swallowing dry food?; Question 5: Do you feel change in saliva viscosity?; Question 6: Do you feel a decreased amount of saliva in your mouth?; Question 7: Do you feel enough or increased amount of saliva in your mouth?; Score: number of positive answers; NE: Not evaluated
